# A Latent Class Modeling Approach to Evaluate Behavioral Risk Factors and Health-Related Quality of Life

**Published:** 2011-10-15

**Authors:** Yongwen Jiang, Matthew M. Zack

**Affiliations:** Center for Health Data and Analysis, Rhode Island Department of Health. Dr Jiang is also affiliated with the Brown University School of Medicine, Providence, Rhode Island; Centers for Disease Control and Prevention, Atlanta, Georgia

## Abstract

**Introduction:**

The Behavioral Risk Factor Surveillance System (BRFSS) monitors multiple health indicators related to 4 domains: risky behaviors, health conditions, health care access, and use of preventive services. When evaluating the effect of these indicators on health-related quality of life (HRQOL), conventional analytical methods focus only on individual risks and thus are not ideally suited for analyzing complex relationships among many health indicators. The objectives of this study were to 1) summarize and group multiple related health indicators within a health domain by using latent class modeling and 2) analyze how 24 health indicators in 4 health domains were associated with 2 HRQOL outcomes to identify Rhode Island adult populations at highest risk for poor HRQOL.

**Methods:**

The 2008 Rhode Island BRFSS, a population-based, random-digit–dialed telephone survey, collected responses from 4,786 adults aged 18 years or older. We used latent class modeling to assign 24 health indicators to high-, intermediate-, and low-risk groups within 4 domains. The effects of all risks on HRQOL were then assessed with logistic regression modeling.

**Results:**

The latent class model with 3 classes fitted the 4 domains best. Respondents with more health conditions and limited health care access were more likely to have frequent physical distress. Those with more health conditions, risky behaviors, and limited health care access were more likely to have frequent mental distress. Use of preventive health services did not affect risk for frequent physical or mental distress.

**Conclusion:**

The latent class modeling approach can be applied to identifying high-risk subpopulations in Rhode Island for which interventions may have the most substantial effect on HRQOL.

## Introduction

The 2 overarching goals of *Healthy People 2010* are "to increase quality and years of healthy life" and "to eliminate health disparities" (1). Tracking population health-related quality of life (HRQOL) can monitor progress toward national health objectives (2) and helps identify health disparities. Besides asking HRQOL questions, population-based health surveys such as the Behavioral Risk Factor Surveillance System (BRFSS) monitor multiple health domains, including risky behaviors, health conditions, health care access, and use of preventive services, which are all associated with HRQOL. Each of these domains has many indicators, most of which are highly correlated. In epidemiologic studies, adjusting for multiple, often correlated, confounders is necessary to draw valid inferences about associations. Because traditional analytical methods such as linear regression (3) or logistic regression (4-12) focus only on individual risks, they are not ideally suited to analyzing complex relationships involving multiple health domains. Indicators measure only special aspects of each domain; therefore, traditional methods must include several indicators to assess these domains comprehensively. However, including highly correlated items can lead to collinearity and problems with estimating and evaluating the association of these domains with outcomes such as HRQOL. This study shows how latent class modeling can summarize indicators within a health domain into latent classes and how logistic regression can then associate these latent classes with HRQOL. Specifically, we applied these methods to Rhode Island 2008 BRFSS data to analyze associations among 24 indicators in 4 health domains with 2 HRQOL outcomes to identify Rhode Island populations at highest risk for poor HRQOL.

## Methods

### Data source

BRFSS is a telephone survey administered in all 50 states and 4 US territories with funding and specifications from the Centers for Disease Control and Prevention (CDC). BRFSS monitors behavioral health risks that contribute to the leading causes of disease and death among adults aged 18 years or older. It also monitors access to health care and certain health conditions. Rhode Island has participated in BRFSS since 1984. From the 2008 Rhode Island BRFSS, a population-based, random-digit–dialed telephone survey of 4,786 respondents, we selected 24 dichotomous indicators of 4 health domains — risky behaviors, health conditions, health care access, and use of preventive services (13) — and 2 HRQOL outcomes. Although CDC's institutional review board (IRB) approved the survey administration, IRB approval was unnecessary for these secondary analyses.

### Indicators

**Figure 1.**
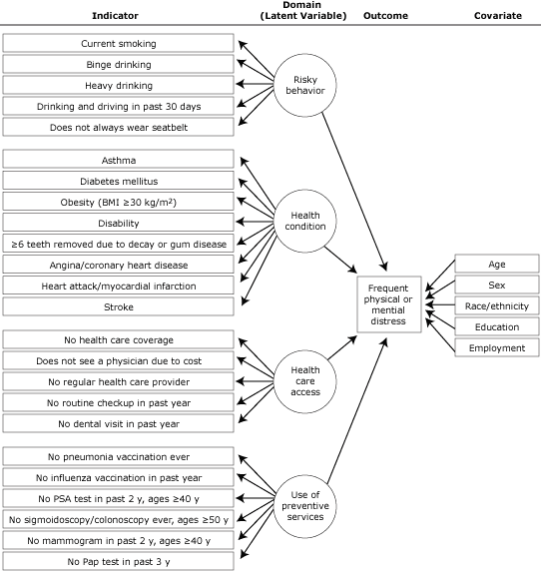
Indicators, health domains, outcomes, and covariates for latent class modeling approach to analyze 2008 data from the Rhode Island Behavioral Risk Factor Surveillance System. Abbreviations: BMI, body mass index; PSA, prostate-specific antigen; Pap, Papanicolaou.

We assessed 5 risky behaviors: current smoking (smokes cigarettes daily or some days), binge drinking (≥5 drinks for men, ≥4 drinks for women on 1 occasion during the past 30 d), heavy drinking (≥3 drinks/d for men, ≥2 drinks/d for women), drinking and driving in the past 30 days, and not always wearing a seatbelt (Figure 1).

Respondents reported ever having had a diagnosis of the following 8 health conditions: asthma (ever being told by a health professional that they had asthma and they have asthma now), diabetes, obesity (body mass index ≥30 kg/m^2^, calculated from self-reported height and weight), disability (limited in any activities or use special equipment), loss of 6 or more teeth because of decay or gum disease, angina/coronary heart disease, heart attack/myocardial infarction, or stroke.

Respondents answered 5 questions about access to health care: being uninsured (no health care coverage), not seeing a physician because of cost (despite needing to, during the past year), having no regular health care provider, no routine checkup in the past year, or no dental visit in the past year.

We assessed use of preventive services with 6 questions: never had a pneumonia vaccination, had no influenza vaccination in the past 12 months, no prostate-specific antigen (PSA) test in the past 2 years (men ≥40 y), never had a sigmoidoscopy or colonoscopy (men and women ≥50 y), had no mammogram in past 2 years (women ≥40 y), or had no Papanicolaou (Pap) test in the past 3 years (women with an intact cervix).

### Outcomes: 2 HRQOL items

The 2 HRQOL items asked respondents about their recent physically unhealthy days ("Now thinking about your physical health, which includes physical illness and injury, for how many days during the past 30 days was your physical health not good?") and mentally unhealthy days ("Now thinking about your mental health, which includes stress, depression, and problems with emotions, for how many days during the past 30 days was your mental health not good?"). These variables were dichotomized at 14 or more days (frequent physical or mental distress) and 0 to 13 days (no physical or mental distress). Others have used this cutpoint to identify frequent distress (5,10).

We included as potential confounders in the multivariable analyses age group (18-44 y, 45-64 y, ≥65 y), sex, race/ethnicity (non-Hispanic white, Hispanic, non-Hispanic other [which includes African Americans because they are rare in the adult Rhode Island population]), education level, and employment status (2,12,14). We excluded income from the models because 14% of respondents lacked income information; we instead used education level and employment status as indicators of socioeconomic status because both highly correlate with income.

### Analyses

The latent class model collapses many discrete indicators into a few meaningful latent classes, categories that refer to levels of a domain, and estimates the probability that a particular person belongs to a specific latent class. The unit of analysis is the response pattern (15,16). In our study, the 5 indicators of risk behaviors have a total of 32 (2^5^) possible response patterns (eg, current smoker vs not current smoker); the 8 indicators of health conditions, 256 (2^8^) possible response patterns; the 5 indicators of access to health care, 32 (2^5^) possible response patterns; and the 6 indicators of use of preventive services, 16 (2^4^) (men) and 32 (2^5^) (women) possible response patterns (17-19). For example, for the 5 indicators of risky behavior, latent class modeling can summarize the 32 possible response patterns into an interpretable number of latent classes (eg, 2-4).

The latent class model provides the prevalence of each latent class (marginal probabilities), and the class-specific response probabilities of each indicator (conditional probabilities) (14,20-23). Formulae and parameters for latent classes appear in the Appendix.

To determine the most parsimonious model, we sequentially fitted models from 1 to more latent classes (16,18,24), compared successive models using the Bayesian information criterion (BIC), and chose the model with the smallest BIC values (25,26) (Table 1).

To examine further the influence of high-risk classes on health, we performed 2 logistic regressions to assess the association of all these latent classes with the 2 outcomes, physical health and mental health. These models estimate odds ratios with 95% confidence intervals for frequent physical and mental distress of the higher risk classes relative to the lower risk classes, adjusting for age, sex, race/ethnicity, education, and employment status.

We used Latent Gold version 4.5 (Statistical Innovations, Inc, Belmont, Massachusetts) for latent class modeling because this software can accommodate the complex sampling design of BRFSS and can also handle missing data. We performed all other analyses with SAS version 9.1 (SAS Institute, Inc, Cary, North Carolina) and accounted for the complex sampling design of BRFSS. Significance was set at *P* < .05.

## Results

**Figure 2.**
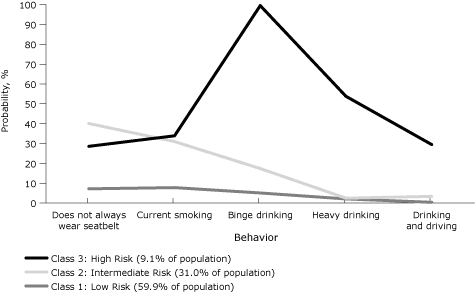
Probabilities of risky behaviors by latent class, Rhode Island Behavioral Risk Factor Surveillance System, 2008. Behaviors refer to the past 30 days. Binge drinking defined as ≥5 drinks for men or ≥4 drinks for women on 1 occasion. Heavy drinking defined as ≥3 drinks/d for men or ≥2 drinks/d for women.

The latent class model with 3 latent classes provided the best fit for all 4 health domains (Table 1). Nine percent of Rhode Island's adult population belonged to the highest risk class for the 5 risky behaviors (Figure 2): 29% or more in this class reported currently smoking cigarettes, binge drinking, heavy drinking, drinking and driving, or not wearing seatbelts. Approximately 31% of the population made up an intermediate class who do not always wear seatbelts, are current smokers, and drink alcohol in binges but do not drink alcohol heavily or drink and drive. In the remaining 60% of the population, less than 8% reported any of these risky behaviors.

**Table:** 

**Behavior**	**Class 3: High Risk (9.1% of Population), %**	**Class 2: Intermediate Risk (31.0% of Population), %**	**Class 1: Low Risk (59.9% of Population), %**
Does not always wear seatbelt	28.6	40.1	7.2
Current smoking	33.9	31.0	7.8
Binge drinking	99.5	17.4	5.1
Heavy drinking	53.8	2.5	2.1
Drinking and driving	29.5	3.4	0.4

**Figure 3.**
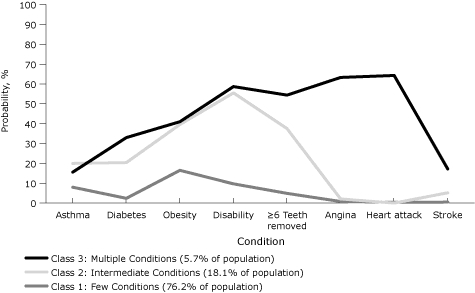
Probabilities of health conditions by latent class, Rhode Island Behavioral Risk Factor Surveillance System, 2008. Health conditions defined as having ever been told by a health professional that they had the condition. Obesity defined as body mass index ≥30 kg/m^2^.

Six percent of the adult population belonged to the highest risk class for the 8 health conditions (Figure 3): at least 15% in this class reported having asthma, diabetes, obesity, a disability, 6 or more teeth lost, angina or coronary heart disease, a myocardial infarction, or a stroke. In 18% of the population, 20% or more reported having asthma, diabetes, obesity, a disability, or 6 or more teeth lost, but less than 6% reported the cardiovascular conditions — angina, heart attack, or stroke. In the remaining three-fourths of the population, 10% or less reported any of these conditions except obesity (16%).

**Table:** 

**Condition**	**Class 3: Multiple Conditions (5.7% of Population), %**	**Class 2: Intermediate Conditions (18.1% of Population), %**	**Class 1: Few Conditions (76.2% of Population), %**
Asthma	15.6	20.0	8.0
Diabetes	33.0	20.4	2.4
Obesity	41.0	39.6	16.5
Disability	58.7	55.5	9.7
≥6 teeth removed	54.4	37.5	4.9
Angina	63.3	2.1	0.8
Heart attack	64.3	0.0	0.4
Stroke	17.2	5.2	0.5

**Figure 4.**
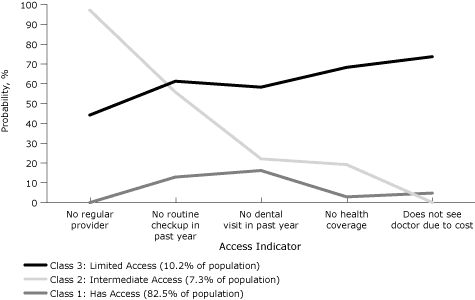
Probabilities of health care access by latent class, Rhode Island Behavioral Risk Factor Surveillance System, 2008.

Ten percent of Rhode Island's adult population belonged to the highest risk class for difficulty accessing the 5 kinds of health care services (Figure 4): at least 45% of these reported having no health care coverage, not being able to see a physician because of cost, having no regular health care provider, having no health checkup in the past year, and having no dental visits in the past year. In 7% of the adult population, at least 78% said they had visited the dentist, had health coverage, and could afford to see a doctor, but at least half had not had a routine checkup in the past year or a regular provider. Less than 16% of the remaining 83% of the adult population reported difficulty accessing any of these kinds of health care.

**Table:** 

**Access Indicator**	**Class 3: Limited Access (10.2% of Population), %**	**Class 2: Intermediate Access (7.3% of Population), %**	**Class 1: Has Access (82.5% of Population), %**
No regular provider	44.2	97.2	0.0
No routine checkup in past year	61.3	55.8	12.9
No dental visit in past year	58.3	22.1	16.2
No health coverage	68.3	19.2	2.9
Does not see doctor due to cost	73.7	0.1	4.8

**Figure 5.**
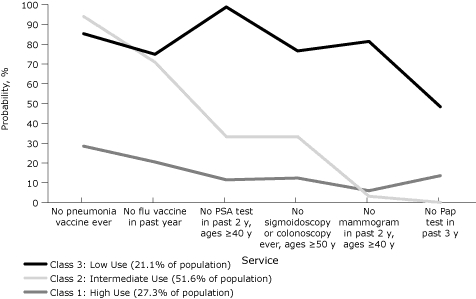
Probabilities of use of preventive services by latent class, Rhode Island Behavioral Risk Factor Surveillance System, 2008. The prostate-specific antigen (PSA) test question was asked only of men. The mammogram and Papanicolaou (Pap) test questions were asked only of women.

Twenty-one percent of Rhode Island's adult population belonged to the highest risk class for not using the 6 kinds of preventive health care services (Figure 5): at least 75% of these had never had a pneumonia vaccination and had not had an influenza vaccination in the past year; at least 77% of those aged 50 or older had never had a sigmoidoscopy or a colonoscopy; 99% of the men aged 40 or older had not had a PSA test in the past 2 years; and at least 48% of women had not had a Pap test in the past 3 years or a mammogram in the past 2 years for women aged 40 or older. In 52% of the population, more than 70% had not had a pneumonia vaccination or an influenza vaccination in the past year but at least 65% had used 1 or more of the other preventive services. In the remaining 27% of the population, at least 70% used all of the different kinds of preventive health care services.

**Table:** 

**Service**	**Class 3: Lower Use (21.1% of Population), %**	**Class 2: Intermediate Use (51.6% of Population), %**	**Class 1: Higher Use (27.3% of Population), %**
No pneumonia vaccine ever	85.3	94.0	28.5
No flu vaccine in past year	74.9	70.9	20.5
No PSA test in past 2 y, ages ≥40 y	98.8	33.3	11.5
No sigmoidoscopy or colonoscopy ever, ages ≥50 y	76.6	33.3	12.4
No mammogram in past 2 y, ages ≥40 y	81.4	3.2	6.0
No Pap test in past 3 y	48.4	0.1	13.6

Approximately 11% of Rhode Island adults reported frequent physical or mental distress. Frequent physical distress was more common in older adults, those with a high school education or less, the retired, the unemployed, and those unable to work (Table 2). Frequent mental distress was more common in younger adults, women, those with some college or less education, homemakers, students, the unemployed, and those unable to work.

Respondents with more health conditions and limited health care access were more likely to have frequent physical distress (Table 3). Those with more risky behaviors, health conditions, and limited health care access were more likely to have frequent mental distress. Use of preventive health services did not affect risk for frequent physical or mental distress.

## Discussion

Our results are consistent with previous findings (8-11,27) that indicators of individual health conditions, such as obesity (8,9) and asthma (27), or individual health care access, such as no health care coverage (11), are associated with frequent physical and mental distress. Individual risky behaviors such as binge drinking (10,11), are only related to frequent mental distress. Our methods allow us to go beyond these results by including many correlated items as indicators for 4 health domains in assessing 2 outcomes. Other authors have either enumerated patterns of adherence to specific health behaviors (28) or summed the number of risk behaviors or health conditions to provide a summary score for analysis (29). The latter approach has been criticized because it weighs risk behaviors or health conditions equally regardless of their severity (30). Latent class modeling is qualitatively more informative than a summary score. For instance, the highest-risk latent class of risky behaviors for Rhode Island adults differs most from lower-risk classes by their larger percentages who binge drink, drink heavily, or drink and drive rather than who do not always use seatbelts or currently smoke.

Combining latent class modeling with logistic regression provides 3 advantages in analyzing data from surveys like BRFSS. First, by grouping multiple correlated health indicators into a few health domains, latent class modeling allows these indicators to become associated with the outcomes through these domains and minimizes multicollinearity. To avoid this problem, many studies (8-11,27) evaluate only some indicators that partially reflect only a specific aspect of each health domain, making their analyses subject to confounding from other excluded indicators. Second, combining latent class models and logistic regression models allows several indicators to contribute to each health domain, thus improving the reliability of these domains by reducing the variation in the health domain due to any single indicator. Finally, once the number of latent classes is selected, latent class models do not require specifying arbitrary cutoffs to distinguish these classes.

These methods analyze men and women together rather than separating them. When analyzed by sex, the patterns of the 4 health domains for men and women were similar. Although some of the indicators in the "preventive services" domain are sex-specific, the Latent Gold software identifies these indicators as structurally missing and treats them as missing at random and totally dependent on the covariates (31). Although men generally use preventive services less often than women, the latent class patterns for the 3 preventive service indicators that are not sex-specific are similar among men and women. Finally, only about 1,690 men and 3,096 women responded to the 2008 Rhode Island BRFSS, making it difficult to carry out sex-specific analyses with so many indicators while avoiding artifacts.

We summarized 24 indicators in 4 health domains to predict 2 outcomes. Some may object that we did not include some important indicators, that some health behaviors were misclassified as health conditions, or that the health domains provide little explanatory power beyond a summary score of risky behaviors and health conditions. BRFSS does not ask the same questions every year. By choosing 2008, we included questions on mammography screening, Pap tests, PSA tests, colorectal screening, oral health, and drinking and driving in the past 30 days. We also excluded questions asked only in odd years about physical activity, eating fruits and vegetables, the burden of arthritis, and gastrointestinal diseases. We excluded questions about HIV and AIDS because very few respondents reported either condition. We also excluded 2 questions on emotional support and life satisfaction that appeared to form a fifth latent health domain because this domain complicated interpretation while not adding much to the 4 existing domains. We included obesity as an indicator for a health condition rather than for a health behavior because we view obesity as an outcome of other behaviors (ie, inadequate diet and physical inactivity) (28).

Besides not being able to include some health indicators, this study has several limitations. BRFSS is cross-sectional, making it difficult to determine whether the risky health behaviors, health conditions, and access to health care increased risk for frequent physical and mental distress or whether this distress increased risk for these behaviors, conditions, and health care access. Because BRFSS respondents reported information, not being able to validate this information and possible recall bias may have affected the observed associations. Latent class modeling has its own limitations that require interpretation. Each class should have a substantial number of observations because small classes may be artifactual outliers. The latent classes themselves should be interpretable in light of current theory and other empirical evidence about the domain they represent.

Despite these limitations, other surveys with multiple health indicators such as the Youth Risk Behavior Survey and the Pregnancy Risk Assessment Monitoring System may benefit from the combined use of latent class modeling and logistic regression in analyzing outcomes of interest. The methods developed here can be applied to other outcomes (eg, diabetes, asthma) and subpopulations (eg, racial/ethnic minorities, people with disabilities).

We conducted separate latent class models for the 4 domains because incorporating all the risk indicators into 1 latent class model was too complicated to allow model convergence. Exploring larger models would be worthwhile. Moreover, when the probability of a pattern of indicators does not differ substantially for different latent classes within a domain, these patterns do not distinguish these latent classes; thus, their labels may not accurately describe all their patterns. For example, those who currently smoke but do not indulge in of any of the other 4 risky behaviors (4% of the sample) are classified into the low-risk class because more in this class have this pattern (42%) than in the intermediate-risk class (35%) and high-risk class (23%).

This study has identified high-risk subpopulations in Rhode Island where health service and health policy interventions may improve HRQOL — among residents with high probabilities of risky behaviors, multiple health conditions, or limited health care access.

## Figures and Tables

**Table 1:** Fit of Latent Class Models Using Bayesian Information Criterion (BIC)^a^ for Health Domains of the Rhode Island Behavioral Risk Factor Surveillance System, 2008

No. of Classes in Model	Health Domain, BIC Value			

	Risky Behavior	Health Condition	Health Care Access	Preventive Service Use
1	17019.17	23700.50	19911.77	18451.38
2	16142.51	22412.98	18306.64	17836.85
3	16119.40	22318.90	18171.87	17661.99
4	16130.11	22354.41	18180.73	17698.54

a Models were sequentially fitted from 1 to more latent classes (16,18,24) and compared to successive models using BIC. The smallest BIC values (25,26) indicate the most parsimonious model.

**Table 2:** Prevalence of Frequent Physical and Mental Distress, by Demographic Characteristics, Rhode Island Adults, Behavioral Risk Factor Surveillance System, 2008

**Characteristic**	No. of Respondents, % (n = 4,786)^a^	Frequent Physical Distress, % (95% CI) (n = 635)	Frequent Mental Distress, % (95% CI) (n = 500)
**Age, y**			
18-44	1,331 (48)	7.2 (5.4-9.0)	12.2 (9.7-14.7)
45-64	1,966 (33)	12.0 (10.3-13.6)	10.4 (8.9-11.9)
≥65	1,446 (18)	18.1 (15.9-20.3)	7.0 (5.5-8.4)
**Sex**			
Male	1,690 (48)	9.4 (7.7-11.1)	8.8 (6.6-11.0)
Female	3,096 (52)	12.0 (10.6-13.5)	12.2 (10.6-13.8)
**Race/ethnicity**			
Non-Hispanic white	4,148 (86)	10.5 (9.3-11.8)	10.4 (9.0-11.9)
Hispanic	320 (9)	13.1 (9.1-17.1)	11.6 (7.1-16.0)
Non-Hispanic other	251 (5)	11.0 (7.0-15.1)	11.4 (6.5-16.3)
**Education**			
High school or less	1,826 (37)	15.8 (13.6-18.1)	13.9 (11.3-16.4)
Some college	1,115 (25)	9.9 (7.7-12.0)	12.0 (9.2-14.7)
College graduate	1,829 (38)	6.6 (5.3-7.9)	6.5 (4.8-8.3)
**Employment status**			
Employed	2,554 (61)	5.9 (4.6-7.1)	8.3 (6.9-9.8)
Homemaker/student	376 (11)	7.2 (3.5-10.8)	14.6 (8.1-21.1)
Retired	1,253 (16)	17.3 (14.9-19.6)	6.1 (4.6-7.7)
Unemployed	271 (7)	17.3 (11.6-23.0)	19.2 (11.9-26.5)
Unable to work	318 (4)	56.2 (49.4-63.0)	35.2 (28.8-41.7)

Abbreviation: CI, confidence interval.

a Some categories do not add to the total because of missing responses. Percentages are weighted.

**Table 3:** Odds Ratios of Frequent Physical and Mental Distress, by Health Domain, Rhode Island Adults, Behavioral Risk Factor Surveillance System, 2008^a^

Health Domain	Frequent Physical Distress		Frequent Mental Distress	

	OR (95% CI)	*P* Value	OR (95% CI)	*P* Value
**Risk behavior**				
Low risk	1 [Reference]	NA	1 [Reference]	NA
Intermediate risk	1.21 (0.88-1.65)	.24	2.32 (1.68-3.21)	<.001
High risk	1.16 (0.47-2.89)	.75	3.85 (2.13-6.98)	<.001
**Health condition**				
Few conditions	1 [Reference]	NA	1 [Reference]	NA
Intermediate conditions	2.40 (1.69-3.41)	<.001	2.52 (1.74-3.64)	<.001
Multiple conditions	3.14 (2.13-4.62)	<.001	2.34 (1.41-3.87)	<.001
**Health care access**				
Has access	1 [Reference]	NA	1 [Reference]	NA
Intermediate access	0.99 (0.54-1.83)	.98	0.86 (0.44-1.68)	.66
Limited access	2.10 (1.14-3.86)	.02	2.40 (1.38-4.17)	.002
**Use of preventive service**				
High use	1 [Reference]	NA	1 [Reference]	NA
Intermediate use	1.05 (0.74-1.49)	.77	1.24 (0.84-1.83)	.27
Low use	1.01 (0.67-1.53)	.95	0.98 (0.62-1.54)	.92

Abbreviations: OR, odds ratio; CI, confidence interval; NA, not applicable.

a Analyses were adjusted for age, sex, race/ethnicity, education, employment status, and all other latent variables in the table. Frequent physical or mental distress was defined as reporting 14 or more physically or mentally unhealthy days in the last 30 days.

## Appendix: Parameters for Latent Classes

For a latent class model with 3 categorical indicators (c1, c2 and c3) and 5 indicators (Y1, Y2, Y3, Y4, Y5), the posterior probability of class c  under pattern of y1, y2, y3, y4 and y5 is

where

= P(y1|c)P(y2|c)P(y3|c)P(y4|c)P(y5|c)

Therefore we need to calculate P(c) and P(y_k_|c) first.

P(c) can be calculated from parameters of *Model for Clusters* as

, c=1,2,3

When Y_k_ is a binary indicator, with 0 for not present and 1 present, *P*(*y_k_* | *c*) can be calculated from parameters for *Models for Indicators* and *Overall Intercepts*, ie,

, k=1,2,3,4,5; c=1,2,3.

**1. Parameters for risk behaviors**

*Model for Clusters*

**Table:**

c1	c2	c3
γ1	γ2	γ3
0	−0.6589	−1.8837

*Models for Indicators,* β_kc_, k = 1 to 5; c = 1 to 3

**Table:**

Risk behavior	Cluster		
	c1	c2	c3
Does not always wear seatbelt	0	2.1580	1.6457
Current smoking	0	1.6739	1.8032
Binge drinking	0	1.3563	8.1758
Heavy drinking	0	0.1468	3.9802
Drinking and driving	0	4.3792	6.8685

*Overall Intercepts*

**Table:**

Does not always wear seatbelt	α1	−2.5588
Current smoking	α2	−2.4719
Binge drinking	α3	−2.9152
Heavy drinking	α4	−3.8289
Drinking and driving	α5	−7.7384

**2. Parameters for health conditions**

*Model for Clusters*

**Table:**

c1	c2	c3
γ1	γ2	γ3
0	−1.4362	−2.5869

*Models for Indicators*, β_kc_, k = 1 to 8; c = 1 to 3

**Table:**

Health indicators	Cluster		
	c1	c2	c3
Asthma	0	1.0616	0.7619
Diabetes	0	2.3319	2.9862
Obesity	0	1.2044	1.2616
Disability	0	2.4585	2.5869
≥6 Teeth removed	0	2.4463	3.1331
Angina	0	0.9534	5.3237
Heart attack	0	−2.5108	6.0319
Stroke	0	2.3855	3.7092

*Overall Intercepts*

**Table:**

Asthma	α1	−2.4489
Diabetes	α2	−3.6929
Obesity	α3	−1.6249
Disability	α4	−2.2358
≥6 Teeth removed	α5	−2.9565
Angina	α6	−4.7777
Heart attack	α7	−5.4432
Stroke	α8	−5.2806

**3. Parameters for health care access**

*Model for Clusters*

**Table:**

c1	c2	c3
γ1	γ2	γ3
0	−2.0890	−2.4236

*Models for Indicators*, β_kc_, k = 1 to 8; c = 1 to 3

**Table:**

Health care acccess	Cluster		
	c1	c2	c3
No regular provider	0	7.5553	11.3359
No routine checkup in past year	0	2.3722	2.1454
No dental visit in past year	0	1.9787	0.3852
No health coverage	0	4.2871	2.0795
Unable to see a doctor because of cost	0	4.0284	−4.3380

*Overall Intercepts*

**Table:**

No regular provider	α1	−7.7890
No routine checkup in past year	α2	−1.9135
No dental visit in past year	α3	−1.6427
No health coverage	α4	−3.5175
Unable to see a doctor because of cost	α5	−2.9970

**4. Parameters for preventive service**

*Model for Clusters*

**Table:**

c1	c2	c3
γ1	γ2	γ3
0	−0.6369	−0.8973

*Models for Indicators*, β_kc_, k = 1 to 8; c = 1 to 3

**Table:**

Preventive service	Cluster		
	c1	c2	c3
No pneumonia vaccine ever	0	−3.6641	−0.9908
No flu vaccine in past year	0	−2.2458	0.1996
No PSA test in past 2 years, ages ≥40	0	−1.3415	5.1220
No sigmoidoscopy or colonoscopy ever, ages ≥50	0	−1.2612	1.8784
No mammogram in past 2 years, ages ≥40	0	0.6606	4.8812
No Pap test in past 3 years	0	5.4541	7.2361

*Overall Intercepts*

**Table:**

No pneumonia vaccine ever	α1	2.7465
No flu vaccine in past year	α2	0.8924
No PSA test in past 2 years, ages ≥40	α3	−0.6957
No sigmoidoscopy or colonoscopy ever, ages ≥50	α4	−0.6932
No mammogram in past 2 years, ages ≥40	α5	−3.4063
No Pap test in past 3 years	α6	−7.3014
